# Query-Specific Deep Embedding of Content-Rich Network

**DOI:** 10.1155/2020/5943798

**Published:** 2020-08-25

**Authors:** Yue Li, Hongqi Wang, Liqun Yu, Sarah Yvonne Cooper, Jing-Yan Wang

**Affiliations:** ^1^School of Economics and Management, Harbin University of Science and Technology, Harbin 150080, China; ^2^The University of Edinburgh, Edinburgh EH8 9JS, Scotland, UK; ^3^New York University Abu Dhabi, Abu Dhabi, UAE

## Abstract

In this paper, we propose to embed a content-rich network for the purpose of similarity searching for a query node. In this network, besides the information of the nodes and edges, we also have the content of each node. We use the convolutional neural network (CNN) to represent the content of each node and then use the graph convolutional network (GCN) to further represent the node by merging the representations of its neighboring nodes. The GCN output is further fed to a deep encoder-decoder model to convert each node to a Gaussian distribution and then convert back to its node identity. The dissimilarity between the two nodes is measured by the Wasserstein distance between their Gaussian distributions. We define the nodes of the network to be positives if they are relevant to the query node and negative if they are irrelevant. The labeling of the positives/negatives is based on an upper bound and a lower bound of the Wasserstein distances between the candidate nodes and the query nodes. We learn the parameters of CNN, GCN, encoder-decoder model, Gaussian distributions, and the upper bound and lower bounds jointly. The learning problem is modeled as a minimization problem to minimize the losses of node identification, network structure preservation, positive/negative query-specific relevance-guild distance, and model complexity. An iterative algorithm is developed to solve the minimization problem. We conducted experiments over benchmark networks, especially innovation networks, to verify the effectiveness of the proposed method and showed its advantage over the state-of-the-art methods.

## 1. Introduction

### 1.1. Background

Recently, content-rich network analysis has attracted much attention. Different from the traditional network, whose each node is only identified by its ID, the content-rich network has content for each node [1, 2]. For example, in a scientific article citation network, each node is a research paper, and each linkage is a citation between two papers, while each node is enriched by the content of the research paper. In this case, each node is represented by not only the paper ID, but also its content, such as its title, abstract, and text. However, in the past researches, the content of each node is ignored and only the network structure is considered to represent the nodes. For example, a popular network analysis tool is network embedding, where each node is mapped to a low-dimensional vector space, where the network structure is preserved [3–6]. The traditional network embedding methods only consider the network structure by learning from the edges of the network, while the content of the nodes is not encoded to the embedding process [7–10]. However, in many cases, the contents of two nodes have a strong clue of the linkage of two nodes, even though there is no direct edge between such two nodes in the network structure alone. As an example, in the innovation network analysis problem, two research papers recently published may have similar ideas, but they have not cited each other. However, from the content of these two papers, we can conclude that they should be sharing the same idea. Thus the content is a good complementary component for the network embedding besides the network structure itself.

Meanwhile, information retrieval is a major application of network analysis. Given a query node in the graph, the search task is to rank the other nodes according to the similarity between the query and the nodes and return the top-ranked nodes [11–15]. Using both the network structure and the query information to rank the nodes in the network has been a popular way for information retrieval, while network embedding is another important direction of network analysis. It is natural to combine these two technologies to improve the performance of the retrieval. However, up to now, all the network embedding works have not considered the query information to boost the embedding for the retrieval results. In this paper, we fill this gap of learning network embeddings for a given specific query and the content of the nodes.

### 1.2. Related Works

In this section, we summarize the related works of network embedding and network-based retrieval. Our work is a query-specific network embedding method that embeds a network for the purpose of searching similar nodes of a given query node. Thus, our work is related to both the network embedding and network-based retrieval works. The related network embedding works are summarized as follows.

  He et al. [2] developed the Network-to-Network Network Embedding model to combine the network structure and content of nodes into one embedding vector. To this end, two neural networks are employed, one for the content based on the convolutional neural network (CNN) model [16–18] and another for the network structure based on the graph convolutional network (GCN) model [19–22]. The CNN model embeds the content of each node to a convolutional representation vector and then feed it to the GCN model, where the representation vectors of neighbors of each node are taken as input and converted to a vector of node identity. The learning of the parameters is performed by minimizing the loss of the node identity prediction task.
  Zhu et al. [6] proposed embedding a node in a network as a Gaussian distribution and using a Wasserstein distance to measure the dissimilarity between two nodes' Gaussian embedding. Moreover, they developed an encoder-decoder method to map the neighborhood coding vector to the Gaussian embedding parameters and then map it back to the neighborhood coding vector. The embedding parameters are optimized to minimize the decoding error and keep the network structures.
  Tu et al. [23] proposed a deep recurrent neural network-based network (RNN) embedding method. It uses a node's neighbors in the network as the input of an RNN model and uses the output of the RNN model to approximate the embedding of the node. The inputs of the neighboring nodes are also their embedding vectors accordingly. Moreover, the RNN outputs are further fed to a multilayer perception (MLP) model to approximate the degree of the node. The learning processes are conducted by minimizing the approximation errors of both the embedding vectors and the degrees.
  Wang et al. [24] innovated a novel graph embedding method for a group of networks. This method tries to learn a group of base vectors, and each vector can extend to a base affinity matrix of a base network by self-product. Then each network affinity matrix is approximated by a learning combination of the base matrices. The learning of the base vectors and the combination coefficients are learned jointly by minimizing the approximation error.

The network-based retrieval works are summarized as follows.

  Li et al. [25] proposed adjusting the affinity matrix of a network according to a given query and a set of positive/negative nodes from the network. This method firstly calculates a ranking vector from the affinity matrix and query node indicator vector and then impose the positive nodes ranking score is larger than the negative nodes. Please note that the positive nodes are the known nodes that are similar to the query, while the negative nodes are the nodes known to be dissimilar to the query node. The learning of the new affinity matrix is conducted by minimizing the loss of the positive/negative nodes constraint and meanwhile keeping the adjusted affinity matrix as similar to the original affinity matrix as possible.
  Yang et al. [26] proposed learning an improved affinity matrix of a network by firstly calculating the tensor product of the matrix and then conducting confusion over the tensor product of the affinity matrix. The tensor product of the matrix is an extended network where each node is a pair of nodes of the original network and each edge weight is the product of the weights of the edges between the corresponding two nodes. The confusion of a matrix is calculated as the summation of the different orders of the matrix and the number of orders varies from zero to infinite. The learned affinity matrix is obtained by recovering from the confusion of the tensor product. They proved that the recovered matrix can be obtained by an iterative algorithm, as *Q* ⟵ *AQA*^*⊤*^+ *I*, where *A* is the original affinity matrix and *Q* is the expected recovered matrix.
  Bai et al. [27] proposed to learn the ranking scores of nodes in a network to a query node by an iterative label prorogation algorithm. In an iterative algorithm, the ranking score of a node is updated as the weighted average of the neighboring nodes, while, at the beginning of each iteration, the ranking score of the query node itself is updated as one.

### 1.3. Our Contributions

Our contribution in this paper is of the following folds:

1. We come up with a novel problem for network analysisthe query-specific content-rich network embedding problem. The setting of this problem is that each node of the network is attached to some content, such as text and image. Moreover, a node or more nodes are known as the query node(s).The task is to learn effective embedding vectors of the nodes so that, from the embeddings, we can calculate a similarity measure to rank the nodes of the network for the purpose of information retrieval.
2. We develop a novel solution to this problem. We use a CNN model to extract the content-level features of each node and then use a GCN model to encode the features of the neighboring nodes to represent the node. The new representations of the nodes by GCN are further converted to the Gaussian distribution parameters, including the mean and the covariance for each node, by an encoder. Finally, the Gaussian distribution parameters are decoded to a node identity probability vector. To learn the parameters, we model the learning problem by minimizing the loss of node identity decoding and network structure-preserving. Meanwhile, to utilize the query node, we try to define a set of positives that are supposed to be relevant to the query and returned by the retrieval system and a set of negative nodes, which is supposed to be ignored by the retrieval system. The labels of the positives/negatives are used to learn the distance between nodes.
3. We design an optimization algorithm to solve the problem of the minimization problem modeled as above. Firstly, the labels of positive and negatives are based on the distance of Gaussian distributions of each pair of nodes measured by the Wasserstein distance, and an upper bound/lower bound of the distance. Secondly, the learning of the parameters of CNN, GCN, Gaussian distribution parameters, and upper bound/lower bound of the labels are learned jointly. Thirdly, learning and labeling are conducted iteratively in an algorithm.

**Remark 1 .**

Our work is based on the idea of learning a probabilistic model relying on an autoencoder architecture, which is well known in the literature as the variational autoencoder (VAE) proposed by Kingma and Welling [28]. Our cost function is different from the standard loss function used in VAEs.

### 1.4. Origination

Our paper is organized as follows. In Section 2, we introduce the novel method for the query-specific network embedding for the content-rich network. In Section 3, we evaluate the proposed method experimentally and compare it against the state-of-the-art. In Section 4, we give the conclusion of this paper.

## 2. Proposed Method

In this section, we will introduce our query-specific embedding method of a content-rich network. The embedding of nodes of the network is conducted at two layers. The first layer is the representation of the content of each node by using the convolutional representation method. The second layer is the representation of the node neighborhood by using graph convolutional representation of the content of the neighboring nodes, where the embedding of the nodes is in the Wasserstein space. To learn the parameters, of the model, we consider the problem of query-specific search, while keeping the network structure.

### 2.1. Content-Rich Graph Embedding

#### 2.1.1. Convolutional Representation of Node Content

In this subsection, we will discuss the presentation of the node contents. Given a node of the network, *v*, we assume its content is a text, which can be denoted as a sequence of words, and each word is represented as an embedding vector:

(1) $$\begin{array}{c} v\;=\;\left\{x_{1},\ldots ,x_{N}\right\}, \end{array}$$

where *x*_*i*_  ∈  *R*^*d*^ is the embedding vector of the *i*-th word, *d* is the dimension of the word embedding space, and *N* is the number of words in the text of node *v*. To represent the text, we employ a CNN model with one convolutional layer and a max-pooling layer.

In the convolutional layer, we have a filter bank, *F* = {*f*_1_,…, *f*_|*F*|_}, where *f*_*k*_ ∈  *R*^(*d* × *s*)^ is the *k*-th filter, which filters the word embeddings of a window size of *s* words. The response of the *k*-th filter is calculated as follows:

(2) qi,k= ReLUf k⊤xi:i+s−1+ bk where k = 1,⋯ ,F and i = 1,⋯ ,N−s+1,

where *x*_*i*:*i*+*s*−1_ ∈  *R*^(*d* × *s*)^ is the concatenation word embedding vectors of *x*_*i*_,…, *x*_*i*+*s*−1_, and *b*_*k*_is the bias parameter of the k-th parameter. Re*LU*(*x*) = max(*x*, 0) is the activation function of rectified linear unit (Re*LU*) [29–32].

In the max-pooling layer, the maximum response of each filter is selected as the output of the layer:

(3) $$\begin{array}{c} \;{\hat{q}}_{k}=\;\max\left(q_{1,k},\ldots q_{\left(\left|v\right|-s\right),k}\right),\;k\;=\;1,\ldots ,\left|F\right|. \end{array}$$

The convolutional representation of the content of the node *v* is the vector of the max-pooling outputs of the |*F*| filter outputs:

(4) $$\begin{array}{c} u\;={\left[{\hat{q}}_{1},\ldots ,\;{\hat{q}}_{\left|F\right|}\right]}^{⊤}\in \;R^{\left|F\right|}. \end{array}$$

#### 2.1.2. Neighborhood Convolutional Encoder-Decoder

In this subsection, we will introduce the neighborhood representation of a node from the content of its neighboring nodes. To this end, we apply an encoding-decoding methodology to code the neighborhood of each node to the Wasserstein space.

*(1) Graph Convolutional Encoder*. We assume the network is denoted as

(5) $$\begin{array}{c} G\;=\;\left(V,E\right),\text{ where }V\;=\;\left\{v_{1},\ldots ,v_{n}\right\}, \end{array}$$

is the set of nodes, *v*_*i*_ is the *i*-th node, and *n* is the number of nodes. *E* = {1,0}^*n*×*n*^ is the matrix of edges, where

(6) $$\begin{array}{c} e_{ij}=\left\{\begin{array}{cc} 1, & \text{if }v_{i}\text{ and }v_{j}\text{ are connected,}\\ 0, & \text{otherwise}. \end{array}\right. \end{array}$$

Thus, we can denote the set of neighbors of a node *v*_*i*_  as *N*_*i*_= {*v*_*j*_|*e*_*ij*_ = 1 or *e*_*ji*_= 1,  *j* = 1,…, *n*}. To represent the neighborhood of a node, we normalize the edge weights of its neighbors as

(7) $$\begin{array}{c} {\hat{e}}_{ij}=\left\{\begin{array}{cc} \frac{\;1+e_{ij}}{1+\sum_{v_{j^{\prime }}\in N_{i}}e_{ij^{\prime }}}, & \text{if }vj\in \;N_{i},\\ 0, & \text{otherwise}. \end{array}\right. \end{array}$$

To utilize the neighborhood to represent a node, we employ the deep graph convolutional network (GCN). The input layer of the GCN is the convolutional representations of nodes, {*u*_1_,…, *u*_*n*_}, where *u*_*i*_ is the representation of content of the *i*-th node,

(8) $$\begin{array}{c} v_{i}^{0}=\;u_{i}.\; \end{array}$$

For the *l*-th layer of GCN, the output is calculated as

(9) $$\begin{array}{c} v{\;}_{i}^{l+1}\;=\tanh\left(W{\;}^{l}\left(\underset{j\in N_{i}}{\sum }{\hat{e}}_{ij}v{\;}_{j}^{l}\;\right)+\;b^{l}\;\right), \end{array}$$

where *v* _*j*_^*l*^ is the input of the *l*-th layer of the *j*-th node and the neighboring nodes' content representations are linearly combined with normalized edge weights and then pass through a full-connection layer with a tanh activation layer, and *W* ^*l*^ and *b*^*l*^ are weight and bias parameters. The number of GCN layers is *L*, and the output of the last layer of GCN for the *i*-th node is denoted as *v* _*j*_^*L*^.

*(2) Gaussian-Based Encoder*. We further assume that each node is generated from a lower-dimensional Gaussian distribution in the Wasserstein space. The Gaussian distribution is characterized as

(10) $$\begin{array}{c} v_{i}∼\;N\;\left({µ}_{i},\Sigma_{i}\right),\; \end{array}$$

where *µ*_*i*_ ∈  *R*^*g*^  is the mean of the distribution, Σ_*i*_ ∈  *R*^*g*×*g*^ is the covariance matrix of the distribution, and *g* is the dimension of the embedding of the lower-dimensional Gaussian distribution. In our work, we assume that the covariance matrix is a diagonal matrix:

(11) $$\begin{array}{c} \Sigma_{i}=\text{ diag }\left(\sigma_{i}\right),\text{ where }\sigma_{i}\in \;R^{g}.\; \end{array}$$

To bridge the network structure and the distribution of the node, we assume that the mean and covariance can be reconstructed from the GCN network output, by two full-connection layers:

(12) $$\begin{array}{c} {µ}_{i}=\;\Theta v{\;}_{i}^{L}+\;\theta ,\;\\ \sigma i=\text{ Elu}\left(\Psi v{\;}_{i}^{L}+\;\psi \right)+\;1, \end{array}$$

where Θ,  Ψ are the weight matrix, while *θ* and *ψ* are the bias vectors.

(13) $$\begin{array}{c} \text{Elu}\left(x\right)\;=\left\{\begin{array}{cc} x, & \text{if }x>\;0,\\ \alpha \;\times \;\left(e^{x}-\;1\right), & \text{if }x\;\leq \;0, \end{array}\right. \end{array}$$

is the Elu (exponential linear unit) activation function [33–36] and Elu(*x*) + 1 is used to guarantee that *σ*_*i*_ is a positive vector. In this way, each node is encoded to a Gaussian distribution in Wasserstein space.

To measure the dissimilarity between the two nodes, *vi* and *vj*, from their Gaussian distributions, we apply the 2nd Wasserstein distance, as follows:

(14) $$\begin{array}{c} \text{dist}\left(vi,vj\right)=\;W_{2}\;\left(N\;\left({µ}_{i},\Sigma_{i}\right),N\;\left({µ}_{j},\Sigma_{j}\right)\right)\\ =\;\left({\left\|{µ}_{i}-\;{µ}_{j}\right\|}_{2}^{2}+{\left\|\Sigma {\;}_{i}^{\left(1/2\right)}\;-\;\Sigma {\;}_{j}^{\left(1/2\right)}\right\|}_{2}^{2}\right). \end{array}$$

*(3) Node-Identity Decoder*. After we have the Gaussian-based encoding result for each node, we want to decode it to its original identity in the graph. Thus, we design a decoder to convert its Gaussian distribution to the probabilities of being the nodes of G. In the decoder, we first sample the data from the Gaussian distribution to obtain a representation of the node, as follows:

(15) $$\begin{array}{c} z_{i}=\;{µ}_{i}+\;\sigma_{i}\times \;\psi ,\psi \;∼\;N\left(0,I\right), \end{array}$$

where *ψ* is the sampled weight parameter. Then we calculate the reconstructed node probability function by a full-connection layer and a sigmoid activation layer.

(16) ui= ReLU Ωzi+ ω,pi= sigmoidΥui+ υ ∈ 0,1n,

where the *j*-th dimension of *p*_*i*_, *p*_*ij*_ is the probability of the node being the *j*-th node of the network.

### 2.2. Problem Modeling and Solving

To learn the parameters of the CNN, GCN, and Gaussian-based encoder-decoder, we consider the following problems.

#### 2.2.1. Decoder Loss of Node Identification

Since the Gaussian-based encoder-decoder is designed to identify the node from the graph, we propose to minimize the loss of the node identification measured the by cross-entropy loss as follows:

(17) $$\begin{array}{c} \min\;\sum_{i=1}^{n}\sum_{j=1}^{n}\pi_{ij}\log\;\left(p_{ij}\right), \end{array}$$

where *π*_*ij*_=1 if *v*_*i*_ is the *j*-th node, and 0 otherwise.

#### 2.2.2. Neighborhood Structure Preservation

With the new coding of each node as a Gaussian distribution in the Wasserstein space, we hope the neighborhood structure can be preserved. To this end, we firstly define a set of triplets of nodes, *T* = {(*i*, *j*, *k*)*|e*_*ij*_= 1, *e*_*ik*_ = 0,  *i*, *j*, *k* = 1,…, *n*}, where *v*_*i*_ and *v*_*j*_ are connected, and *v*_*i*_ and *v*_*k*_ are disconnected in graph *G*. The energy between two nodes *v*_*i*_ and *v*_*j*_ in the graph is also defined as the Wasserstein distance:

(18) $$\begin{array}{c} \varpi_{ij}=\text{ dist}\left(v_{i},v_{j}\right). \end{array}$$

To keep the structure of the network, we propose minimizing the squared energy between the connected nodes and maximizing the exponential of negative entity between the disconnected nodes:

(19) $$\begin{array}{c} \min\;\sum_{\left(i,j,k\right)\in T}\left(\varpi_{\;ij}^{2}+\;\exp\left(-\varpi_{ik}\right)\right). \end{array}$$

With minimizing this objective, we hope the learned Gaussian distributions of the connected nodes are close in the Wasserstein space, while that of the disconnected nodes are far from each other in the Wasserstein space. Thus, the network structure is preserved in the Wasserstein space.

#### 2.2.3. Query-Specific Distance Supervision

In our problem setting, we already have a known query node, and the task is to find similar nodes in the network. We assume the *q*-th node is the query node, *v*_*q*_. To use the query to guild the learning process, we define a label for each node to indicate if it is similar to the query. By default, *y*_*q*_ is similar to itself; thus, *y*_*q*_= 1. For the other nodes, it is difficult to define the label accurately. Thus, we develop a heuristic method to learn the labels from the Wasserstein distance between the query node and a given candidate node. For this purpose, we split the distance range to three intervals, divided by an upper bound, *u*, and a lower bound, *l*, where *l*  <  *u*. The labeling process selects the nodes which have a Wasserstein distance to query larger than *u* as positives and selects the nodes with a Wasserstein distance to query smaller than *l* as negative. The nodes whose distance to query is between *u* and *l* are left to be ambiguous. Thus the label of a node *vi* is defined as

(20) $$\begin{array}{c} yi=\left\{\begin{array}{cc} 1, & \text{ if }i\;=\;q,\\ 1,\; & \text{if dist}\left(v_{i},v_{q}\right)\leq \;l,\text{ and }i\;\neq \;q,\\ -1,\; & if\text{ dist }\left(vi,vq\right)\;\geq \;u\text{ and }i\;\neq \;q,\\ \text{None}, & \text{otherwise}. \end{array}\right. \end{array}$$

We further define an indicator to indicate if *v*_*i*_ is labeled as *β*_*i*_= 1 and 0 otherwise. The range of distance between *u* and *l* is the range of ambiguous nodes, and we define *u*  −  *l* as the ambiguous range. For the labeled nodes, we minimize a linear loss of *L*(*v*_*i*_, *v*_*j*_; *y*_*i*_)= *y*_*i*_ ×  dist(*v*_*i*_, *v*_*q*_). Meanwhile, we also hope the ambiguous range can be as small as possible so that more nodes can be labeled. Thus, we minimize *u*  −  *l*. The overall minimization problem is the combination of both the minimization of the linear losses of the labeled and the ambiguous range:

(21) $$\begin{array}{c} \begin{array}{cc} \min & \overset{n}{\underset{i=1}{\sum }}\beta_{i}y_{i}\text{ dist }\left(v_{i},v_{q}\right)\;+\;\gamma \left(u\;-\;l\right)\\ \text{ s}.\text{t}.\; & 0\;\leq \;l\;\leq \;u, \end{array} \end{array}$$

where *γ* is a regularization parameter. In this way, for the positive nodes which are labeled to be similar to the query, their distance to the query should be minimized, while the distance to the query for the negatives will be maximized.

The overall optimization problem is the combination of the three subproblems:

(22) $$\begin{array}{c} \begin{array}{cc} \min{\;}_{\Phi ,u,l}\;o\left(\Phi ,u,l\right) & =\left\{\overset{n}{\underset{i=1}{\sum }}\overset{n}{\underset{i=1}{\sum }}\pi_{ij}\;\log\left(p_{ij}\right)+C_{1}\underset{\left(i,j,k\right)\in T}{\sum }\left(\varpi_{\;ij}^{2}+\exp\left(-\varpi_{ik}\right)\right)+C_{2}\left(\overset{n}{\underset{i=1}{\sum }}\beta_{i}y_{i}\text{dist}\left(v_{i},v_{q}\right)\;+\;\gamma \left(u\;-\;l\right)\right)+\;C_{3}\left\|\Phi \right\|{\;}_{2}^{2}\right.\\ \text{s}.\text{t}.\; & 0\;\leq \;l\;\leq \;u, \end{array} \end{array}$$

where Φ represents the set of parameters of CNN, GCN, and Gaussian-based encoder-decoder. Solving this problem directly is difficult, because the label definition, ambiguous range parameters, and the Gaussian-based encoder parameter are coupled. To be specific, the label is defined over the ambiguous range and the distance of the Gaussian-based distributions of the nodes, while the parameters of the Gaussian-based distributions are learned from the labels of the nodes. To solve this problem, we use the fixed point iteration method [37–40] in an iteration algorithm.

We firstly fix the parameters of Φ, and the ambiguous range parameters, *u* and *l*, to update the labels according to (20).

Then we fix the labels and ambiguous range parameters to update the parameters of Φ by solving the following problem:

(23) $$\begin{array}{c} \min{\;}_{\Phi }\;o_{1}\left(\Phi \right)\;=\;\left\{\sum_{i=1}^{n}\sum_{i=1}^{n}\pi_{ij}\;\log\left(p_{ij}\right)\right.\;+C_{1}\underset{\left(i,j,k\right)\in T}{\sum }\left(\varpi_{\;ij}^{2}+\exp\left(-\varpi_{ik}\right)\right)\left.\;+\;C_{2}\left(\overset{N}{\underset{i=1}{\sum }}\beta_{i}y_{i}\text{ dist }\left(v_{i},v_{q}\right)\;\right)+\;C_{3}{\left\|\Phi \right\|}_{2}^{2}\right\}. \end{array}$$

This problem is solved by the back-propagation algorithm with the ADAM optimizer [41].

Finally, we fix Φ and the labels to update the ambiguous range parameters as follows:

(24) $$\begin{array}{c} \begin{array}{cc} {\text{min}}_{u,l} & o_{2}\left(u,l\right)=\;C_{2}\gamma \left(u\;-\;l\right)\\ \text{s}.\text{t}. & 0\;\leq \;l\;\leq \;u. \end{array} \end{array}$$

We use the gradient descent algorithm to solve this problem:

(25) $$\begin{array}{c} u\;⟵\;u\;-\;\rho \frac{\partial o_{2}\left(u,l\right)}{\partial u}\;,\\ l\;⟵\;l\;-\;\rho \frac{\partial o_{2}\left(u,l\right)}{\partial l},\; \end{array}$$

where *ρ* is the descent step size. Since (∂*o*_2_(*u*, *l*)/∂*u*)=1 and (∂*o*_2_(*u*, *l*)/∂*l*)=−1, in each descent step, *u* is increased by *ρ*, while *l* is decreased by *ρ*, until *u* = *l*.

## 3. Experimental Results

In this section, we conduct experiments over benchmark data sets of networks.

### 3.1. Datasets

In the experiments, we use the following benchmark datasets of the innovation networks.

  The first dataset is Cora dataset [42]. This dataset is a network of research articles of machine learning topics. The research articles are treated as nodes, and the edges are the citations of papers. The abstract of each article is treated as the content of each node. This network has only 2,211 nodes and 5,214 edges. The content of articles has around 170 words on average; the total number of unique works of nodes in this network is 12,619.
  The second dataset is Citeseer dataset [43]. This dataset is a network of scientific articles of ten different multidisciplinary topics. Each node is also a research article, and each edge is the same citation relation connecting two articles. But in this network, the content of each node is its title, not the abstract. The number of nodes of this network is 4,610, and the number of edges is 5,923. The number of words of content is 10 on average, and the number of unique words overall is 5,523.
  The third dataset is the DBLP dataset [44]. This network has the bibliography data of 13,404 articles of computer science. Each node is an article, and each edge is a citation relation. The articles are labeled by four different research topics, including artificial intelligence and computer vision. The content of each node is also the title. The number of edges is 39,861. The average length of the content is 10, and the size of the unique word set is 8,501.

### 3.2. Experimental Settings

To conduct the experiments, we set up the following protocols by using the leave-one-out validation. For each network, we leave one node out as a query node, and the remaining nodes as the candidate nodes to be retrieved. Since our data is research articles, we define the relevance of two research articles according to their small areas. If an article is in the same small areas as the query article, then it is defined as a positive node. The task is to retrieve as many positives as possible while keeping the negatives out of search results as much as possible. This process is repeated for each node of the network by turns; that is, each node is treated as a query node one by one. Then we apply our algorithm to learn the embeddings of the nodes and use the Wasserstein distances to measure the dissimilarity between the query and a candidate node and rank them according to returning the nodes with the smallest Wasserstein distances. To measure the performance of the retrieval results, we use the mAP (mean average precision) [45, 46].

**Remark 2 .**

*mAP* is an effective measure of database retrieval performance. Given a set of queries, *Q*, the retrieval systems return a list of ranked database objects for each query. For each query, *q*  ∈  *Q*, we can calculate a precision at each rank *k*:

(26) $$\begin{array}{c} \text{precision }\left(q\right)@k\;=\frac{TP\;\left(q\right)@k\;}{k}\;,\; \end{array}$$

where *TP*(*q*)@*k* is the number of return objects relevant to *q* at top *k* ranks. The average precision (*AP*) of *q* is calculated as the average overall ranks:

(27) $$\begin{array}{c} AP\left(q\right)\;=\;\frac{1\;}{K}\;\sum_{k=1}^{K}\text{precision }\left(q\right)@k,\; \end{array}$$

while *mAP* is the mean of *AP* over the queries at *Q*.

(28) $$\begin{array}{c} mAP\;=\;\frac{1\;}{\left|Q\right|}\;\sum_{q\in Q}AP\left(q\right),\; \end{array}$$

where |*Q*| is the size of *Q*.

### 3.3. Experimental Results

We compare the proposed method, named as Query-Specific Deep Embedding of Content-Rich Network (QDECN), against the network-based ranking methods first and then against the other network embedding methods. For the comparison to the network embedding methods, we first embed the nodes of the network and then calculate the dot-product scores of their embedding vectors and the query as the ranking scores for the purpose of retrieval.

#### 3.3.1. Comparison with Network-Based Ranking Methods

We compared the following methods of network-based ranking: graph transduction (GT) [27], tensor product graph diffusion (TPGD) [26], and Query-Specific Optimal Networks (QUINT) [25]. The comparison results are shown in Table 1. From this table, we can see that the proposed method QDECN outperforms the other methods in all cases. This is not surprising at all due to the following reasons.

1. QDECN is the only method that explores the content of the nodes of a network, while, for all the other methods, they only utilize the network structure information, such as edge data. However, in these datasets of innovation networks, two articles may not have the citation relation, but according to their content similarity, they should belong to the same small area. QDECN not only codes the content of the nodes to its representation but also leverage the content features of its neighboring nodes. So it has the capability to learn from both the node content and the edges of the network, while the other network-based ranking methods only learn from the network structure itself.
2. QDECN is the only method that employs network embedding technology to improve retrieval performance. The remaining methods, such as QUINT and TPGD, aim to learn a better network affinity matrix to guild the learning of the ranking score, but they are still failing to the same schema as GT. The network-based ranking methods use the network affinity matrix to regularize the learning of ranking score, but the network embedding methods map the nodes to a low-dimensional continues vectors, which contains the richer information about the network and instinct information about the relevance of nodes; thus, it is a better choice for the node relevance search tasks.
3. Only QDECN and QUINT adjust the learning of the network parameters according to the query node. This method can learn a better network representation which is optimal for finding the relevant nodes to the node. This setting does not guarantee the learned network representation is optimal for other tasks, but, for the given query node, it gives better results than other methods. Please also note that the supervision information of QUINT is richer than QDECN, since the positive/negative nodes of QUINT are given as ground truth, but for QDECN the positives and negatives are both learned by the algorithm. However, QDECN still outperforms QUINT in all cases.

#### 3.3.2. Comparison to Network Embedding Methods

We compare QDECN to the following network embedding methods: Network to-Network Network Embedding (Net2Net-NE) [2], Deep Variational Network Embedding in Wasserstein Space (DVNE) [6], and Deep Recursive Network Embedding (DRNE) [23]. The results are shown in Table 2. We have the following observations from this table.

1. Again, QDECN obtains better results than the other methods. Compared to DRNE and DVNE, the proposed method and Net2Net-NE can use the content of the nodes to enrich the embedding results. Compared to the Net2Net-NE itself, our method can further sense which node is the query node and take this advantage to guild the embedding process, which Net2Net-NE cannot. Due to the above reasons, the overall results of QDECN are better than the others.
2. DRNE and DVNE are common network embedding methods, which have no supervisor of node content and query node. Meanwhile, Net2Net-NE has the supervision from the content of the node but cannot access the query. Thus, this is not a fair competition. However, our method is the very first algorithm that can use both the node content and query node of the networks. The fact that QDECN gives the best results is a piece of strong evidence that it is necessary to develop an effective method to take both node content and query node into account during the process of network embedding, especially for the purpose of information retrieval.

**Remark 3 .**

To obtain the results of Tables 1 and 2, we set the values of the parameters as follows: for Cora dataset, *C*_1_= 100,  *C*_2_= 100,  *C*_3_= 100; for Citeseer dataset, *C*_1_= 10,  *C*_2_= 10,  *C*_3_= 100; and for DBLP,  *C*_1_= 100,  *C*_2_= 100,  *C*_3_= 100. There are two dimensionalities of the latent spaces, *d* and *g*, and we set their values to 300 and 500 for three datasets. The learning rate of the optimizer is set to 0.01 for all three datasets.

### 3.4. Parameter Analysis

In our model, there are three tradeoff parameters, *C*_1_,  *C*_2_,  and *C*_3_. We conduct experiments to analyse them one by one.

#### 3.4.1. Analysis of *C*_1_

**Figure 1:**
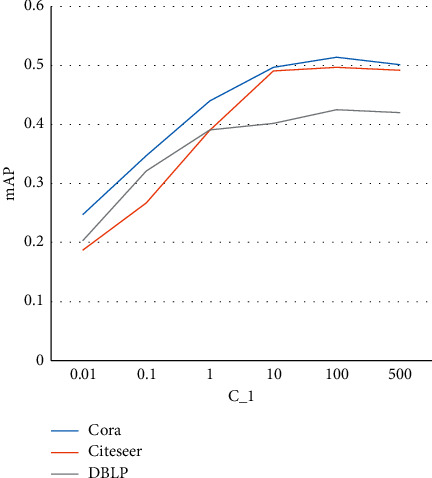
Sensitivity analysis of *C*_1_.

*C*_1_ is the weight of the network structure preservation loss term. We vary the value of *C*_1_ and measure the changes of *mAP* over three different datasets and plot the curves in Figure 1. We can see that the performance of our algorithm keeps improving when the value of *C*_1_ is increased. Since this parameter is the weight of the network neighborhood structure preservation term, this phenomenon indicates that the neighborhood structure plays an important role in a good quality network embedding process and also is critical for the node-level information relevance search problem. Moreover, we also observe that performance improvement becomes minor after a certain value. For example, for the DBLP network, this value is 1, while, for the Citeseer network, this value is 10.

#### 3.4.2. Analysis of  *C*_2_

**Figure 2:**
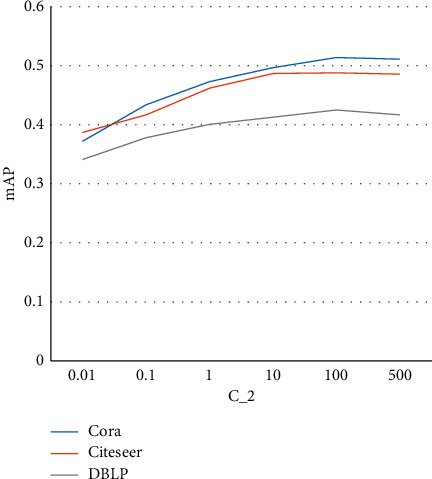
Sensitivity analysis of *C*_2_.

*C*_2_ is the weight of the loss term of the supervision of positive/negatives nodes regarding the Wasserstein distance. The performance curves of different *C*_2_ values are shown in Figure 2. From this figure, we can also conclude that overall a larger value of *C*_2_ can give a better performance in terms of mAP. But the improvement is limited, and the performance is not sensitive to the change of the values of *C*_2_. A possible reason is that the positive and negative labeling is not at the level of ground truth but is estimated by the upper/lower bound. However, the upper/lower bound itself is learned as variable. Thus, in nature, the learning process is an unsupervised learning process. So the improvement is not comparable to supervised learning. Even though it is unsupervised, we still can see the improvements with increasing *C*_2_, which is the benefit from the sensing of the query node.

### 3.5. Computational Intensity

In this section, we study how computational intensive the proposed method is. The average running time (in seconds) of the learning process for a query over the datasets of Cora, Citeseer, and DBLP is 43.66, 93.34, and 437.04. The running time is rather long because the model needs to be retrained for each query node, and the neighborhood preserving term in (19) scale as n_3_ as we are considering all possible triples within the network. This problem is even more serious with the network being large. Thus, we proposed to reduce the size of training triplet set size. To this end, for each node, instead of using all the disconnected nodes to construct the training triplets, we only sample a few disconnected node for this purpose. After this change, the running time is reduced to 21.12, 55.07, and 94.34, respectively.

### 3.6. Contribution of Different Objective Terms during the Training Phase

**Figure 3:**
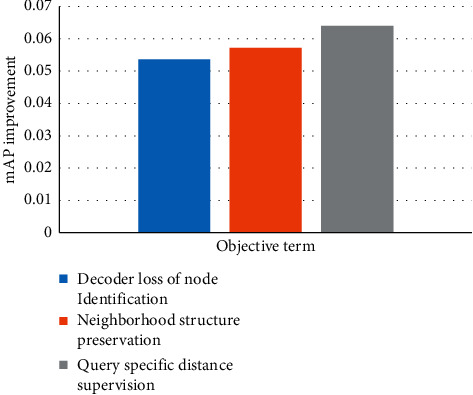
Term-wise mAP improvement.

In this section, we analyse the contributions of the different terms of the objective for the database of Cora. To measure the contribution of a term, we firstly remove this term from the objective and learn the model to retrieve the nodes for queries and calculate the mAP of the retrieval results. Then we add the term back to the objective and measure the retrieval results by mAP again. The contribution of this term is measured by the improvement of the mAP after the term is added to the objective. The term-wise mAP improvement is reported in Figure 3. From this figure, we can see that the query-specific distance supervision term gives the largest contribution, while the regularization term has the least contribution. The second and third significant contributions are from the node identification term and the neighborhood structure preservation term.

## 4. Conclusions

In this paper, we develop a novel method of network embedding, for the content-rich network, for the purpose of node-level information retrieval. We firstly use a CNN to extract features from the content and then use a GCN to code the features of the neighboring nodes, and finally use a deep encoder-decoder to map these features to a Gaussian distribution and convert it to the node's identity. The learning of the parameters is performed by minimizing a loss function. In the loss function, except for the node identification loss, the neighborhood preservation loss, and the complexity of models, we also consider the query node regularization problem. For this purpose, we define positive/negative nodes according to the Wasserstein distance between the query and the candidate nodes. Experimental results show the advantages of the proposed method which embeds the content-rich network guided by the query node.

## Figures and Tables

**Figure 4:**
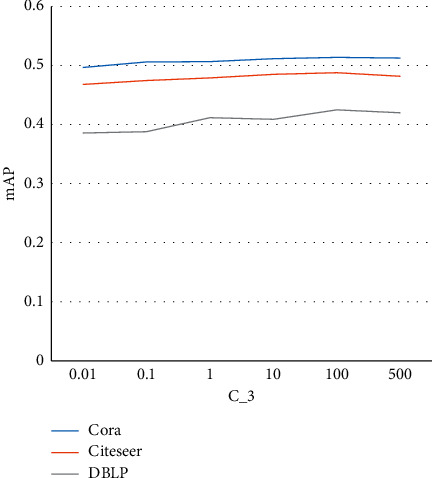
Sensitivity analysis of *C*_3_.

**Table 1:** Comparison of results of network-based ranking methods.

*y*	GT	TPGD	QUINT	QDECN
Cora	0.428	0.431	0.486	0.514
Citeseer	0.394	0.406	0.461	0.497
DBLP	0.355	0.361	0.377	0.425

**Table 2:** Comparison of results of network embedding methods.

Dataset	DRNE	DVNE	Net2Net-NE	QDECN
Cora	0.430	0.433	0.492	0.514
Citeseer	0.403	0.418	0.477	0.497
DBLP	0.397	0.403	0.413	0.425

## Data Availability

All the data sources used in this work to produce the experimental results are available online.

## Appendix

### Sensitivity to Parameter *C*_3_

The tradeoff parameter *C*_3_ is the weight of the squared *ℓ*_2_ norms of the parameters of models to control the complexity of the models. The changes of the performances of the algorithm with different values of *C*_3_ are shown in Figure 4. It is shown that the performance remains stable with the changing *C*_3_. The algorithm is insensitive to this parameter.

## References

[1] Chen H., Sharp B. M. (2004). Content-rich biological network constructed by mining pubmed abstracts. *BMC Bioinformatics*.

[2] He Z., Liu J., Li N., Huang Y. Learning network-to-network model for contentrich network embedding.

[3] Tang J., Qu M., Wang M., Zhang M., Yan J., Mei Q. Line: large-scale information network embedding.

[4] Wang D., Cui P., Zhu W. Structural deep network embedding.

[5] Wang X., Cui P., Wang J., Pei J., Zhu W., Yang S. (2017). Community preserving network embedding. *Proceedings of the Thirty-First AAAI Conference on Artificial Intelligence*.

[6] Zhu D., Cui P., Wang D., Zhu W. Deep variational network embedding in wasserstein space.

[7] Gao H., Pei J., Huang H. Progan: network embedding via proximity generative adversarial network.

[8] Jin D., Rossi R. A., Koh E., Kim S., Rao A., Koutra D. Latent network summarization: bridging network embedding and summarization.

[9] Liu N., Tan Q., Li Y., Yang H., Zhou J., Hu X. Is a single vector enough?: exploring node polysemy for network embedding.

[10] Yang D., Rosso P., Li B., Cudre-Mauroux P. Nodesketch: highly-efficient graph embeddings via recursive sketching.

[11] Dominich S. n., Skrop A. (2005). Pagerank and interaction information retrieval. *Journal of the American Society for Information Science and Technology*.

[12] Geyik S.C., Ambler S., Kenthapadi K. Fairness-aware ranking in search & recommendation systems with application to linkedin talent search.

[13] Hughes J. W., Chang K. H., Zhang R. Generating better search engine text advertisements with deep reinforcement learning.

[14] Liu T. Y. (2009). Learning to rank for information retrieval. *Foundations and Trends R ◯ in Information Retrieval*.

[15] Wang Z., Long C., Cong G., Ju C. Effective and efficient sports play retrieval with deep representation learning.

[16] Chen Y., Xu L., Liu K., Zeng D., Zhao J. Event extraction via dynamic multi-pooling convolutional neural networks.

[17] Dong L., Wei F., Zhou M., Xu K. Question answering over freebase with multicolumn convolutional neural networks.

[18] Long M., Cao Y., Cao Z., Wang J., Jordan M. I. (2018). Transferable Representation Learning with Deep Adaptation networks. *IEEE Transactions on Pattern Analysis and Machine Intelligence*.

[19] Chiang W. L., Liu X., Si S., Li Y., Bengio S., Hsieh C. J. Cluster-gcn: an efficient algorithm for training deep and large graph convolutional networks.

[20] Gao H., Pei J., Huang H. Conditional random field enhanced graph convolutional neural networks.

[21] Ma Y., Wang S., Aggarwal C. C., Tang J. Graph convolutional networks with eigenpooling.

[22] Zügner D., Günnemann S. Certifiable robustness and robust training for graph convolutional networks.

[23] Tu K., Cui P., Wang X., Yu P. S., Zhu W. Deep recursive network embedding with regular equivalence.

[24] Wang S., Arroyo J., Vogelstein J. T., Priebe C. E. (2019). Joint Embedding of Graphs. *IEEE Transactions on Pattern Analysis and Machine Intelligence*.

[25] Li L., Yao Y., Tang J., Fan W., Tong H. Quint: on query-specific optimal networks.

[26] Yang X., Prasad L., Latecki L. J. (2013). Affinity learning with diffusion on tensor product graph. *IEEE Transactions on Pattern Analysis and Machine Intelligence*.

[27] Bai X., Yang X., Latecki L. J., Liu W., Tu Z. (2010). Learning context-sensitive shape similarity by graph transduction. *IEEE Transactions on Pattern Analysis and Machine Intelligence*.

[28] Kingma D. P., Welling M. (2013). Auto-encoding variational bayes. https://arxiv.org/abs/1312.6114.

[29] Agarap A. F. (2018). Deep learning using rectified linear units (relu). https://arxiv.org/abs/1803.08375.

[30] Dahl G. E., Sainath T. N., Hinton G. E. Improving deep neural networks for lvcsr using rectified linear units and dropout.

[31] Hara K., Saito D., Shouno H. Analysis of function of rectified linear unit used in deep learning.

[32] Nair V., Hinton G. E. Rectified linear units improve restricted Boltzmann machines.

[33] Clevert D. A., Unterthiner T., Hochreiter S. (2015). Fast and accurate deep network learning by exponential linear units (elus). https://arxiv.org/abs/1511.07289.

[34] Li Y., Fan C., Li Y., Wu Q., Ming Y. (2018). Improving deep neural network with multiple parametric exponential linear units. *Neurocomputing*.

[35] Shah A., Kadam E., Shah H., Shinde S., Shingade S. Deep residual networks with exponential linear unit.

[36] Trottier L., Gigu P., Chaib-Draa B. Parametric exponential linear unit for deep convolutional neural networks.

[37] Ishikawa S. (1974). Fixed points by a new iteration method. *Proceedings of the American Mathematical Society*.

[38] Martinez-Yanes C., Xu H.-K. (2006). Strong convergence of the cq method for fixed point iteration processes. *Nonlinear Analysis: Theory, Methods & Applications*.

[39] Popova E. D. Generalization of a parametric fixed-point iteration.

[40] Rhoades B. E. (1976). Comments on two fixed point iteration methods. *Journal of Mathematical Analysis and Applications*.

[41] Kingma D. P., Ba J. (2014). Adam: a method for stochastic optimization. https://arxiv.org/abs/1412.6980.

[42] McCallum A. K., Nigam K., Rennie J., Seymore K. (2000). Automating the construction of internet portals with machine learning. *Information Retrieval*.

[43] Lim K. W., Buntine W. (2016). Bibliographic analysis with the citation network topic model. https://arxiv.org/abs/1609.06826.

[44] Tang J., Zhang J., Yao L., Li J., Zhang L., Su Z. Arnetminer: extraction and mining of academic social networks.

[45] Henderson P., Ferrari V. End-to-end training of object class detectors for mean average precision.

[46] Li K., Huang Z., Cheng Y. C., Lee C. H. A maximal figure-of-merit learning approach to maximizing mean average precision with deep neural network based classifiers.

